# First high quality draft genome sequence of a plant growth promoting and cold active enzyme producing psychrotrophic *Arthrobacter agilis* strain L77

**DOI:** 10.1186/s40793-016-0176-4

**Published:** 2016-08-26

**Authors:** Ram N. Singh, Sonam Gaba, Ajar N. Yadav, Prakhar Gaur, Sneha Gulati, Rajeev Kaushik, Anil K. Saxena

**Affiliations:** 1 Division of Microbiology, ICAR-Indian Agricultural Research Institute, New Delhi, 110012 India; 2 Present Address: ICAR-National Bureau of Agriculturally Important Microorganisms, Kushmaur, Mau, 275103 Uttar Pradesh India

**Keywords:** *Arthrobacter*, Psychrotrophic, PGPB, Cold-active enzymes, Pangong Lake, Himalayas

## Abstract

*Arthrobacter agilis* strain L77, is a plant growth promoting and cold active hydrolytic enzymes producing psychrotrophic bacterium, isolated from Pangong Lake, a subglacial lake in north western Himalayas, India. Genome analysis revealed metabolic versatility with genes involved in metabolism and cold shock adaptation, utilization and biosynthesis of diverse structural and storage polysaccharides such as plant based carbon polymers. The genome of *Arthrobacter agilis* strain L77 consists of 3,608,439 bp (3.60 Mb) of a circular chromosome. The genome comprises of 3316 protein coding genes and 74 RNA genes, 725 hypothetical proteins, 25 pseudo-genes and 1404 unique genes.

**Electronic supplementary material:**

The online version of this article (doi:10.1186/s40793-016-0176-4) contains supplementary material, which is available to authorized users.

## Introduction

The microorganisms from extreme environments are of particular importance in global ecology since the majority of terrestrial and aquatic ecosystems of our planet are permanently or seasonally submitted to cold temperatures. Microorganisms capable of coping with low temperatures are widespread in these natural environments where they often represent the dominant flora and they should therefore be regarded as the most successful colonizers of our planet. Members of the genus *Arthrobacter* [1, 2] are Gram-positive, show rods in exponential growth and cocci in their stationary phase, able to grow under aerobic as well as anaerobic conditions and belong to the phylum *Actinobacteria* [3]. Different species of *Arthrobacter* [1, 2] have been implicated in plant growth promotion [4], production of industrially important enzymes [5, 6] and as xeroprotectant [7, 8]. These reports suggest that species from *Arthrobacter* [1, 2] harbor genes for coding enzymes that can be useful in the industry, agriculture and biotechnology. *Arthrobacter agilis* [9] strain L77 was isolated from Pangong Lake, a subglacial lake in north western Himalayas, India and exhibit plant growth promoting attributes as well as production of hydrolytic enzymes. The culture was further characterized for production of EPS and anti-freeze compounds (AFCs). Here, we present the draft genome sequence of *Arthrobacter agilis* [9] strain L77 along with the description of genome properties and annotation.

## Organism information

### Classification and features

*Arthrobacter agilis* [9] strain L77 was isolated from frozen sub-glacial Pangong Lake (33°82′55.59″N and 78°59′26.69″E) in north western Himalaya, India (Table 1). This psychrotrophic bacterium was isolated using standard serial dilution method on Trypticase soya agar [10] plate and has been reported to possess plant growth promoting attributes and could produce cold active enzymes and AFCs. It could solubilize phosphorus, zinc and could produce indole acetic acid and ammonia. It could produce cold active enzymes such as lipase, amylase, protease, chitinase and β-galactosidase.

**Table 1:** Classification and general features of *Arthrobacter agilis* strain L77

MIGS ID	Property	Term	Evidence code^a^
	Classification	Domain *Bacteria*	TAS [12]
		Phylum *Actinobacteria*	TAS [3]
		Class *Actinobacteria*	TAS [13]
		Order *Actinomycetales*	TAS [2, 14]
		Family *Micrococcaceae*	TAS [2, 15]
		Genus *Arthrobacter*	TAS [1, 2]
		Species *Arthrobacter agilis*	TAS [9]
		Strain L77	NAS
	Gram stain	Positive	IDA
	Cell shape	Polymorphic: Coccus to rod shaped	IDA
	Motility	Non-motile	TAS [9]
	Sporulation	Non-sporulating	TAS [9]
	Temperature range	−10 °C −30 °C	IDA
	Optimum temperature	15 °C	IDA
	pH range; Optimum	6–9, 7	IDA
	Carbon source	Yeast extract, glucose, lactose, mannose	TAS [9]
MIGS-6	Habitat	Sub-glacial Lake	IDA
MIGS-6.3	Salinity	Grown on 5 % > NaCl (w/v)	IDA
MIGS-22	Oxygen requirement	Aerobic	TAS [9]
MIGS-15	Biotic relationship	Free living	TAS [9]
MIGS-14	Pathogenicity	Non-pathogeneic	NAS
MIGS-4	Geographic location	India, Leh Ladakh, Jammu & Kashmir	TAS [10]
MIGS-5	Sample collection	March 28, 2010	IDA
MIGS-4.1	Latitude	33°82′55.59″N	NAS
MIGS-4.2	Longitude	78°59′26.69″E	NAS
MIGS-4.4	Altitude	3215 m	NAS

^a^Evidence codes - *TAS* Traceable Author Statement (i.e., a direct report exists in the literature), *NAS* Non-traceable Author Statement (i.e., not directly observed for the living, isolated sample, but based on a generally accepted property for the species, or anecdotal evidence). These evidence codes are from the Gene Ontology project [49]

**Fig. 1:**
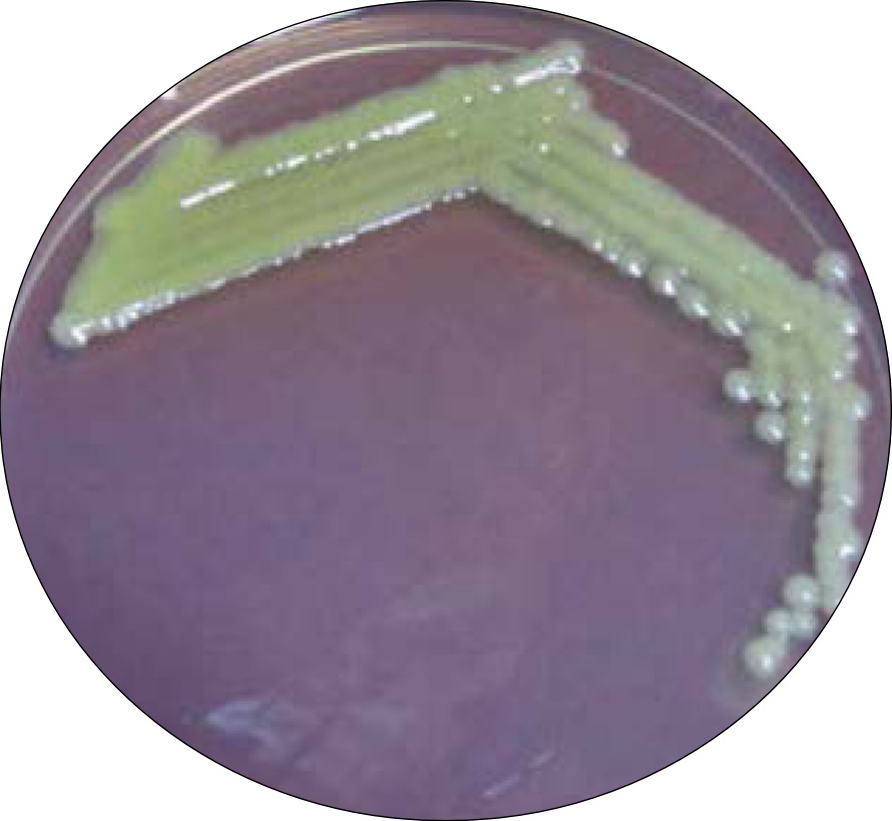
Full grown *yellow* colored bacterial culture on Tripticase Soy Agar (TSA) medium

Strain L77 is a bright yellow colored (Fig. 1) Gram-positive, aerobic, non-motile bacterium exhibiting a rod-coccus cycle. The initial validation of bacterium was done by 16S rRNA gene sequencing using the universal eubacterial primers pA (5′-AGAGTTTGATCCTGGCTCAG-3′) and pH (5′-AAGGAGGTGATCCAGCCGCA-3′) [11]. The 16S rRNA gene sequence places *Arthrobacter agilis* strain L77 in the domain *Bacteria* [12] (Table 1), phylum *Actinobacteria* [3] and Class *Actinobacteria* [13], order *Actinomycetales* [2, 14] and family *Micrococcaceae* [2, 15] during homology search by BLAST [16]. Only few of the closely related species after reclassification [17] of genus *Arthrobacter* [1, 2,] with validly published names: *A. agilis*DSM 20550^**T**^ [9], *A. woluwensis* 1551^**T**^DSM 10495 [18], *A. methylotrophus*DSM 14008^**T**^ [19], *A. tecti*LMG 22282^**T**^ [20], *A. parietis*LMG 22281^**T**^ [20], *A. subterraneus* CH7^**T**^DSM 17585 [21], *A. tumbae*LMG 19501^**T**^ [20], *Arthrobacter oryzae* KV-651^**T**^DSM 25586 [22], *Arthrobacter alkaliphilus* LC6^**T**^DSM 23368 [23], *Arthrobacter flavus*JCM 11496^**T**^ [24], *A. cupressi* D48^**T**^DSM 24664 [25], *A. globiformis*DSM 20124^**T**^ [1, 2] were selected for drawing the phylogenetic position of strain L77.

**Fig. 2:**
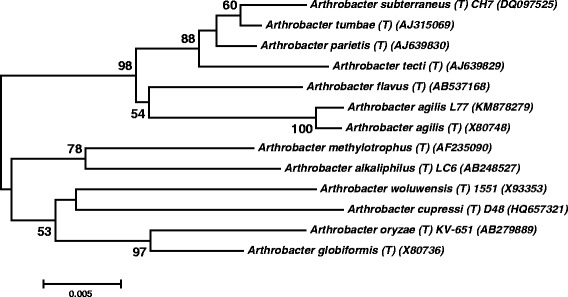
Phylogenetic placements of *Arthrobacter agilis* strain L77 between known species of *Arthrobacter* genus

A phylogenetic tree was constructed (Fig. 2) from the 16S rRNA gene sequence together with other *Arthrobacter* [1, 2] homologs using MEGA 6.0 software suite [26]. The evolutionary history was inferred by using the Maximum Likelihood method based on the Tamura-Nei model [27]. The tree with the highest log likelihood (0.14495825) is shown. The percentage of trees in which the associated taxa clustered together is shown next to the branches. Initial tree(s) for the heuristic search were obtained automatically by applying Neighbor-Join and BioNJ algorithms to a matrix of pairwise distances estimated using the Maximum Composite Likelihood (MCL) approach, and then selecting the topology with superior log likelihood value. The tree is drawn to scale, with branch lengths measured in the number of substitutions per site. The analysis involved 13 nucleotide sequences. All positions containing gaps and missing data were eliminated. There were a total of 1553 positions in the final dataset. Evolutionary analyses were conducted in MEGA6.0 [26]. According to the 16S rRNA gene similarity, the nearest phylogenetic neighbors of *Arthrobacter agilis* strain L77 are *Arthrobacter flavus*JCM 11496^**T**^ [24] (AB537168) with 97.8 %, *A. tecti*LMG 22282^**T**^ [20] (AJ639829) with 97.13 %, *A. parietis*LMG 22281^**T**^ [20] (AJ639830) with 97.41 %, *A. subtrerraneus* CH7^**T**^DSM 17585 [21] (DQ097525) with 97.66 % and *A. tumbae*LMG 19501^**T**^ [20] (AJ315069) with 97.68 % similarity. The 16S rRNA gene sequence also submitted to NCBI GenBank with the accession number KT804924.

### Extended feature descriptions

**Fig. 3:**
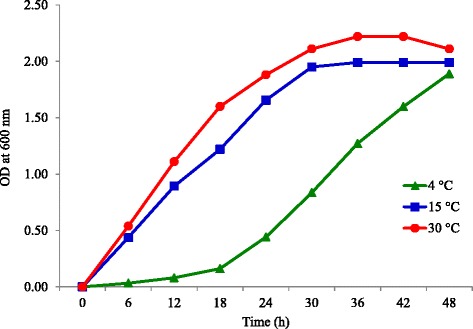
Growth curves of *Arthrobacter agilis* strain L77 at three different temperatures 4, 15 and 30 °C

*Arthrobacter agilis* strain L77, a psychrotrophic bacterium, forms bright yellow color colonies (Fig. 1) on TSA medium and could grow in a pH range of 6–9 and tolerate 5 % NaCl. Growth studies showed that the isolate when incubated at 15 and 30 °C was in the exponential phase until 36 h, while at 4 °C, the exponential phase started after 24 h (Fig. 3). Freezing survival studies of *Arthrobacter agilis* strain L77 revealed that when the culture was initially grown at 4 °C prior to freezing at −10 and −20 °C, it showed significantly higher freezing survival rather than culture initially grown at 15 and 30 °C prior to freezing (Fig. 3).

**Fig. 4:**
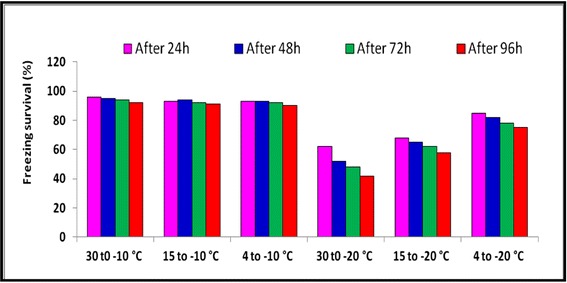
The survival of *Arthrobacter agilis* strain L77 subjected to freezing temperature (−10 and −20 °C) shifted from three different temperatures 4, 15 and 30 °C

Exopolysaccharide production was found to be higher at lower incubation temperatures (4 or 15 °C) in comparison to the optimal growth temperature (30 °C) for *Arthrobacter agilis* (L77) (Fig. 4). EPS production by psychrophilic bacteria is one of the adaptations at low temperatures. The high polyhydroxyl content of EPS lowers the freezing point and ice nucleation temperature of water. In addition, EPS can trap water, nutrients and metal-ions and facilitate surface adhesion, cellular aggregation and biofilm formation and may also play a role in protecting extracellular enzymes against cold denaturation and autolysis [28, 29].

**Fig. 5:**
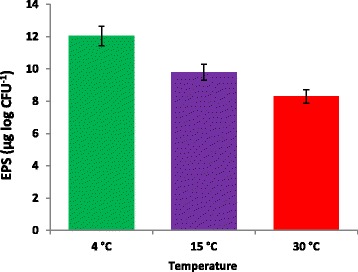
EPS accumulation by *Arthrobacter agilis* strain L77 at three different temperatures 4, 15 and 30 °C

Remarkable variations in terms of accumulation of various organic acids, sugars, polyols and amino acids were detected through HPLC at three different incubation temperatures (4, 15 and 30 °C) (Additional file 1: Table S1, Additional file 2: Table S2 and Fig. 5). Among the sugars, accumulation of mannitol and sorbitol was observed only at 4 °C. The amino acids expression pattern revealed that the most prominent increase was observed in the concentrations of glycine, cysteine and arginine at 4 °C (Additional file 2: Table S2). It has been reported that the cold active enzymes and efficient growth rates are used to facilitate and maintain the adequate metabolic fluxes at near freezing temperature for cold-adaptation [30]. The development of freezing tolerance by producing cryoprotectant compounds or adaptation of cytoplasmic enzymes to cold conditions for protecting cytoplasmic components is one of the strategy used by microbial cells to survive in freezing conditions as these molecules depress freezing point for the protection of cells [31].

Enhanced EPS production by the psychrophilic bacteria at low temperature suggests that EPS plays an important role in desiccation protection or prevention of drying of bacterial cells from freezing temperature. It can be assumed that the strain L77 follows a cold evading strategy to thrive in freezing conditions by synthesizing various cryoprotectants (sugars, polyols and amino acids). These cryoprotectants are known to depress freezing point to evade crystallization [32].

## Genome sequencing information

### Genome project history

This organism was selected for sequencing on the basis of its environmental and agricultural relevance to help in plant growth and ability to provide inorganic phosphate to crops at very low temperature. It also has biogeochemical importance of producing AFCs, so helpful for soil aeration. The genome project is deposited in the online genome database (NCBI-Genome). Sequencing, assembly and annotations were performed at Division of Microbiology, Indian Agricultural Research Institute (ICAR-IARI), New Delhi, India. A summary of the project information is shown in the Table 2.

**Table 2:** Genome sequencing project information for *Arthrobacter agilis* strain L77

MIGS ID	Property	Term
MIGS-31	Finishing quality	Unfinished, improved high quality draft
MIGS-28	Libraries used	Paired End (insert size 250 bp)
MIGS-29	Sequencing platforms	Illumina MiSeq
MIGS-31.2	Fold coverage	180×
MIGS-30	Assemblers	A5 pipeline v jan-2014
MIGS-32	Gene calling method	Prodigal
	Locus Tag	RY94
	Genbank ID	JWSU00000000.1-10.1
	Genbank Date of Release	08-Jan-2015
	GOLD ID	Gp0117366
	BIOPROJECT	PRJNA270909
MIGS 13	Source Material Identifier	L77
	Project relevance	Bioprospecting

### Growth conditions and genomic DNA preparation

A culture of L77 was grown in Trypticase soya broth, until they reached an OD_(600 nm)_ > 1.0. The cells were pelleted from 5 ml culture, washed thrice with TE buffer (10 mM Tris and 1 mM EDTA, pH 8.0) and the pellet was resuspended in 750 μl TE buffer. Genomic DNA was extracted from the suspended pellet using Zymo Research Fungal/Bacterial DNA MicroPrep™ following the standard protocol prescribed by the manufacturer.

### Genome sequencing and assembly

The draft genome of *Arthrobacter agilis* strain L77 (PRJNA270909) was generated at the Division of Microbiology, ICAR-Indian Agricultural Research Institute (ICAR-IARI), New Delhi, India using Illumina [33] technology (Table 2). For this genome, we constructed and sequenced an Illumina MiSeq shotgun library which generated 1,568,654 reads totaling 321.8 Mb data. The raw fastq data was checked for quality using Fast QC [34]. Trimmomatic 0.32 [35] with Nextra adapter sequences was used to hard clip reads. Assembly of trimmed reads was carried out using a5 pipeline version 2014 [36] (Table 2). In terms of N50 and total number of scaffolds, the a5 pipeline [36] was found to be better than other genome assemblers. CONTIGuator [37] was used to improve the assembly draft. The final draft was identified as *Arthrobacter agilis* L77, using megablast with RDP 16S database, release 11–1 [38]. This whole-genome project (Bioproject ID: PRJNA270909) has been registered and assembled sequence data submitted at NCBI GenBank under the accession no. JWSU00000000.1-10.1. The version described in this paper is the first version.

### Genome annotation

Genes were identified using Prokka 1.8 [39] based on Prodigal [40] (Table 2) as part of the Oak Ridge National Laboratory genome annotation pipeline. The predicted CDSs were further annotated on Pfam [41], and (COGs) [42]. These data sources were combined to assert a product description for each predicted protein. Non-coding genes and miscellaneous features were predicted using tRNAscan-SE [43], RNAMMer [44], Rfam [45], TMHMM [46], and signalP v4.1 [47] (Table 3).

**Table 3:** Genome Statistics for *Arthrobacter agilis* strain L77

Attribute	Value	% of total
Genome size (bp)	3,608,439	100.00
DNA coding (bp)	3,224,998	89.37
DNA G + C (bp)	2,518,329	69.79
DNA scaffolds	10	100.00
Total genes	3390	100.00
Protein coding genes	3316	97.81
RNA genes	84	2.18
Pseudo genes	25	0.73
Genes in internal clusters	N/A	N/A
Genes with function prediction	2591	78.10
Genes assigned to COGs	2122	63.64
Genes assigned to Pfam domains	2855	85.11
Genes with signal peptides	126	5.51
Genes with transmembrane helices	852	25.6
CRISPR repeats	N/A	N/A

## Genome properties

**Fig. 6:**
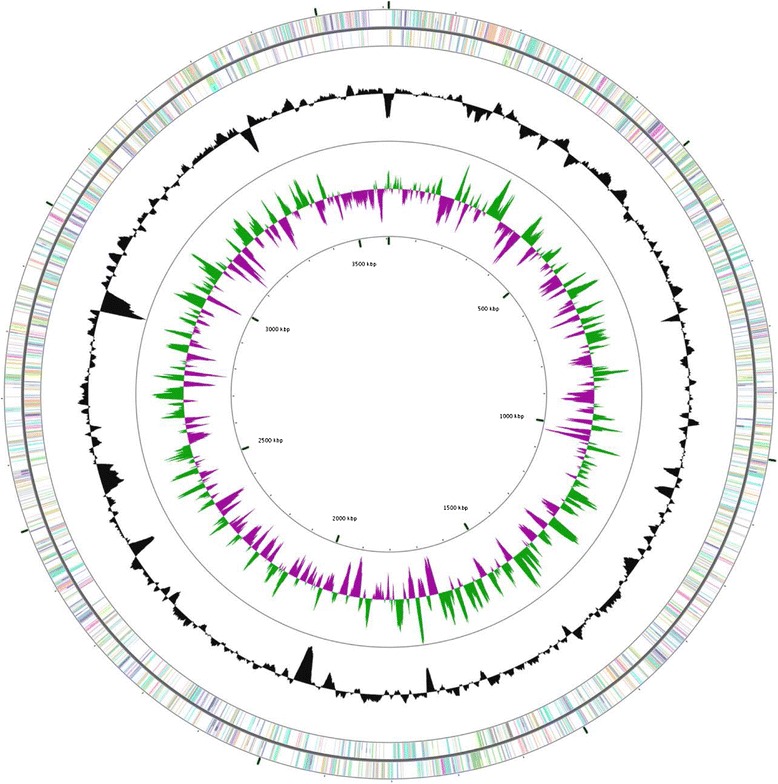
Graphical map of genome of *Arthrobacter agilis* strain L77. From outside to centre: RNA genes (*Brown*, tRNA and *light purple*, rRNA) and other genes are colored according to COG categories. Inner circle shows the GC skew with positive (+) as *dark green* and negative (−) as *dark purple*. GC content is indicated in *black*

The genome is 3,608,439 bp in size, which has GC content of 69.79 mol % (Table 3). There are 47 tRNA, 1 tmRNA, 6 rRNA and 20 ncRNA genes. Of the 3390 predicted genes, 3316 are protein-coding genes (CDSs). Of the total CDSs, 63.64 % represent COG functional categories and 5.51 % consist of signal peptides (Table 3). The distribution of genes into COG functional categories are presented in Table 4. The genome map (Fig. 6) was visualized by CG view server [48].

**Table 4:** Number of protein coding genes of *Arthrobacter agilis* strain L77 associated with general COG functional categories

Code	Value	% age^a^	COG category
J	184	5.54	Translation, ribosomal structure and biogenesis
A	1	0.03	RNA processing and modification
K	208	6.27	Transcription
L	109	3.28	Replication recombination and repair
B	1	0.03	Chromatin structure and dynamics
D	22	0.66	Cell cycle control, Cell division, chromosome partitioning
V	49	1.47	Defense mechanisms
T	113	3.40	Signal transduction mechanisms
M	124	3.73	Cell wall/membrane biogenesis
N	30	0.90	Cell motility
U	19	0.57	Intracellular trafficking and secretion
O	104	3.13	Posttranslational modification, protein turnover, chaperones
C	110	3.31	Energy production and conversion
G	213	6.42	Carbohydrate transport and metabolism
E	200	6.03	Amino acid transport and metabolism
F	71	2.14	Nucleotide transport and metabolism
H	114	3.43	Coenzyme transport and metabolism
I	88	2.65	Lipid transport and metabolism
P	118	3.55	Inorganic ion transport and metabolism
Q	38	1.14	Secondary metabolites biosynthesis, transport and catabolism
R	204	6.15	General function prediction only
S	166	5.00	Function unknown
–	1030	31.06	Not in COGs

^a^The total is based on the number of protein coding genes in the annotated genome

## Insights from the genome sequence

The isolate was successfully screened for lipase, amylase, protease, chitinase and β-galactosidase. Genome analysis showed two important genes *pstA* and *pstC* which are required for the translocation of phosphate across the membranes. Another important gene, PstB (an ADP binding protein), of the phosphate transport system is responsible for giving energy to the phosphate transport system of the organism. PhoR and PhoP were also found which are important for regulation of phosphate operon. PhoH like protein has a probable ATPase which is induced when phosphate level decreases. Genome annotation also predicted a putative cold shock protein which is supposed to play an important role in low temperature conditions. There are other proteins which shares evolutionary relationship with bacterial cold shock proteins such as Rhodanase and S1 RNA binding protein suggesting their role in low temperature conditions. In-depth analysis of the genome could give us better insight into mechanism of tolerance of this strain to low temperature. Other temperature responsive proteins were found such as molecular chaperone Hsp31 and glyoxalase 3 that influence the exposure of hydrophobic domains of proteins and stabilize the early unfolding under high temperature stress conditions to provide stability to the isolate in temperature stress.

Genes of heavy metal resistance were also found in the annotation. Mercuric resistance operon regulatory protein activates the mercury resistance operon in the presence of mercury thus protecting the bacteria from harmful side-effects of mercury. Mercuric reductase is also present which is responsible for conversion of Hg^2+^ to Hg^0^. *copZ* is a copper chaperone that replaces zinc with copper and releases *copY* from the DNA which is a negative regulator of *copYZAB* under excess copper. Gene of nitrogen regulation, nitrogen regulatory protein P-II was found that regulates the level of nitrogen by regulating glutamine. When the ratio of glutamine to 2-ketoglutarate decreases, uridine is added on a tyrosine of P-II to form P-II-UMP which in turn deadenylates glutamine synthase resulting in its activation. Putative genes coding for these activities were identified in the genome based on annotation (Table 5).

**Table 5:** Candidate genes coding for putative lipase, amylase, chitinase, protease, β-galactosidase, phosphate transport regulation, cold shock proteins, chaperons and heavy metal resistance activities identified in *Arthrobacter agilis* strain L77 draft genome

Putative Gene	Annotation	Size (aa)
	Lipase	
ABAGL_00531	GDSL-like Lipase/Acylhydrolase	262
ABAGL_00732	Lipase 1 precursor	288
ABAGL_00875	GDSL-like Lipase/Acylhydrolase	267
ABAGL_01161	Lipase 1 precursor	350
ABAGL_03217	GDSL-like Lipase/Acylhydrolase	272
	Amylase	
ABAGL_00299	Glucose-resistance amylase regulator	338
ABAGL_01452	Glucose-resistance amylase regulator	336
ABAGL_01652	Trehalose synthase/amylase TreS	588
ABAGL_01737	Alpha-amylase precursor	905
ABAGL_01923	Alpha-amylase/pullulanase	257
ABAGL_01950	Glucose-resistance amylase regulator	327
	Chitinase	
ABAGL_01394	putative bifunctional chitinase/lysozyme precursor	520
ABAGL_01777	Chitinase	400
	Protease	
ABAGL_00100	Putative cysteine protease YraA	188
ABAGL_00190	Flp pilus assembly protein, protease CpaA	207
ABAGL_00447	Lon protease	364
ABAGL_00456	Putative serine protease HtrA	496
ABAGL_00667	Serine proteasec	401
ABAGL_00940	CAAX amino terminal protease self- immunity	268
ABAGL_00971	CAAX amino terminal protease self- immunity	247
ABAGL_01091	Serine protease Do-like HtrA	366
ABAGL_01213	Rhomboid protease GluP	291
ABAGL_01289	ATP-dependent zinc metalloprotease FtsH	689
ABAGL_01302	Putative ATP-dependent Clp protease ATP-binding subunit	835
ABAGL_01392	CAAX amino terminal protease self- immunity	266
ABAGL_01505	Minor extracellular protease vpr precursor	1059
ABAGL_01669	Flp pilus assembly protein, protease CpaA	168
ABAGL_01755	CAAX amino terminal protease self- immunity	326
ABAGL_02020	Putative serine protease HtrA	310
ABAGL_02206	Putative metalloprotease	303
ABAGL_02449	Putative zinc metalloproteasec/MT2700	388
ABAGL_02467	Modulator of FtsH protease HflK	310
ABAGL_02638	ATP-dependent Clp protease ATP-binding subunit ClpX	430
ABAGL_02639	ATP-dependent Clp protease proteolytic subunit 1	224
ABAGL_02640	ATP-dependent Clp protease proteolytic subunit 2	208
ABAGL_02862	ATP-dependent Clp protease adaptor protein ClpS	105
ABAGL_02923	ATP-dependent zinc metalloprotease FtsH	438
ABAGL_03163	Serine protease inhibitor-like protein	389
ABAGL_03211	CAAX amino terminal protease self- immunity	267
ABAGL_03271	Metalloprotease MmpA	447
ABAGL_00551	Protease PrtS precursor	355
ABAGL_00739	Protease 2	734
ABAGL_01958	Protease synthase and sporulation negative regulatory protein	215
ABAGL_02571	Protease PrsW	425
ABAGL_03295	Protease 3 precursor	455
	β-galactosidase	
ABAGL_00260	β-galactosidase bgaB	667
ABAGL_00292	β-galactosidase	687
ABAGL_01083	β-galactosidase precursor	708
	Phosphate Transport Regulation	
ABAGL_01317	Phosphate transport system permease protein PstA	310
ABAGL_01318	Phosphate import ATP-binding protein PstB	367
ABAGL_01316	Phosphate transport system permease protein PstC	259
ABAGL_00191	Alkaline phosphatase synthesis sensor protein PhoR	544
ABAGL_03137	Alkaline phosphatase synthesis sensor protein PhoR	555
ABAGL_01671	PhoH-like protein	443
ABAGL_02530	PhoH-like protein	344
	Cold shock Proteins	
ABAGL_01978	putative cold shock protein A	67
	Chaperons	
ABAGL_01554	Molecular chaperone Hsp31 and glyoxalase 3	255
ABAGL_01067	Copper chaperone CopZ	74
	Heavy Metal Resistance	
ABAGL_02628	Mercuric resistance operon regulatory protein	134

## Conclusions

The 3.6 Mb draft genome of *Arthrobacter agilis* strain L77 was assembled and annotated. The isolate was successfully screened for production of EPS and AFCs with potential application in biotechnology. The candidate genes coding for hydrolytic enzymes and cold shock proteins were identified in the genome. *Arthrobacter agilis* strain L77 will serve as a source for antifreeze proteins, functional enzymes and other bioactive molecules in future bioprospecting projects.

## Additional files

Additional file 1: Table S1. Quantitative analysis of organic acid and sugars/polyols from *Arthrobacter agilis* strain L77 by HPLC. (DOCX 13 kb) Additional file 2: Table S2. Quantitative analysis of amino acids content of *Arthrobacter agilis* strain L77 by HPLC. (DOCX 15 kb)

## Abbreviations

AFCs: Anti-freeze compounds
EPS: Exopolysaccharides
