# Supplementary material for: First high quality draft genome sequence of a plant growth promoting and cold active enzyme producing psychrotrophic Arthrobacter agilis strain L77
Source: Stand Genomic Sci. 2016 Aug 26;11(1):54. doi: 10.1186/s40793-016-0176-4 (PMC5000428; doi:10.1186/s40793-016-0176-4)
Supplement: Additional file 1: Table S1. — Quantitative analysis of organic acid and sugars/polyols from Arthrobacter agilis strain L77 by HPLC. (DOCX 13 kb) [file 40793_2016_176_MOESM1_ESM.docx]

**Additional file 1: Table S1.** Quantitative analysis of organic acid and sugars/polyols from *Arthrobacter agilis* strain L77 by HPLC

| S.No | | µg mL^-1^ | | |
| --- | --- | --- | --- | --- |
|  |  | 4 °C | 15 °C | 30 °C |
| Organic acid | |  |  |  |
|  | Gluconic acid | 113.0 ± 2.2 | 60.77 ± 1.4 | 16.73 ± 1.4 |
|  | Citric acid | 120.5 ± 2.5 | 45.16 ± 1.2 | - |
|  | Formic acid | 37.03 ± 1.3 | - | - |
|  | Fumaric acid | 34.43 ± 1.4 | - | - |
|  | Propionic acid | 24.63 ± 1.9 | 15.58 ± 3.4 | 6.19 ± 3.2 |
|  | Succinic acid | 63.48 ± 1.6 | 49.99 ± 1.4 | - |
|  | Tartaric acid | 18.66 ± 1.7 | - | - |
| Sugars/polyols | |  |  |  |
|  | Glucose | - | - | - |
|  | Trehalose | 2.4± 1.6 | 7.4± 1.5 | - |
|  | Raffinose | 25.2±0.5 | 12.2±0.2 | 5.6±1.2 |
|  | Mannitol | 7.5± 1.5 | - | - |
|  | Sorbitol | 12.3± 1.3 | - | - |
