# Supplementary material for: First high quality draft genome sequence of a plant growth promoting and cold active enzyme producing psychrotrophic Arthrobacter agilis strain L77
Source: Stand Genomic Sci. 2016 Aug 26;11(1):54. doi: 10.1186/s40793-016-0176-4 (PMC5000428; doi:10.1186/s40793-016-0176-4)
Supplement: Additional file 2: Table S2. — Quantitative analysis of amino acids content of Arthrobacter agilis strain L77 by HPLC. (DOCX 15 kb) [file 40793_2016_176_MOESM2_ESM.docx]

**Additional file 2: Table S2.** Quantitative analysis of amino acids content of *Arthrobacter agilis* strain L77 by HPLC

| S.No | Amino acids | Code | Picomole mg^-1^cell dry weight^1^ | | |
| --- | --- | --- | --- | --- | --- |
|  |  |  | 4 °C | 15 °C | 30 °C |
|  | Alanine | Ala | 65.8 ± 1.5 | 47.5 ± 0.6 | 39.9 ± 0.9 |
|  | Arginine | Arg | 435.5 ± 9.8 | 238.2 ± 3.9 | 197.5 ± 4.5 |
|  | Aspartic acid | Asp | 32.8 ± 0.7 | 17.4 ± 0.45 | 9.7 ± 2.6 |
|  | Cysteine | Cys | 485.0 ± 10.9 | 219.0 ± 1.4 | 157.9 ± 1.3 |
|  | Glutamic acid | Glu | 111.8 ± 0.3 | 67.4 ± 0.25 | 26.2 ± 0.6 |
|  | Glycine | Gly | 662.0 ± 14.9 | 533.3 ± 12.2 | 347.5 ± 7.5 |
|  | Histidine | His | 97.6 ± 2.2 | 55.6 ± 1.35 | 12.6 ± 4.3 |
|  | Isoleucine | Iso | 158.1 ± 3.6 | 89.4 ± 0.25 | 27.6 ± 0.6 |
|  | Leucine | Leu | 190.5 ± 4.3 | 140.0 ± 16 | 108.3 ± 24 |
|  | Lysine | Lys | 97.5 ± 1.3 | 66.3 ± 1.5 | 57.73± 2.5 |
|  | Methionine | Meth | 130.2 ± 0.7 | 85.5± 1.5 | 46.5 ± 1.2 |
|  | [Ornithine](http://en.wikipedia.org/wiki/Ornithine) | [Orn](http://en.wikipedia.org/wiki/Ornithine) | 97.1 ± 0.6 | 66.5 ± 0.12 | 24.9 ± 1.5 |
|  | Phenylalanine | Phe | 51.6 ± 0.3 | 22.4 ± 0.3 | 17.50 ±2.5 |
|  | Proline | Pro | 240.0 ± 0.9 | 123.9 ± 2.7 | 51.6 ± 0.9 |
|  | Serine | Ser | 76.3 ± 0.45 | 66.5 ± 1.3 | 54.4 ± 1.2 |
|  | Threonine | Thr | 19.7 ± 0.25 | 6.7 ± 0.22 | 2.5 ± 0.4 |
|  | Tyrosine | Tyr | 21.2 ± 0.5 | 14.9 ± 0.12 | 8.6 ± 1.8 |
|  | Tryptophan | Try | 48.3 ± 0.25 | 38.0 ± 0.4 | 11.7 ± 1.2 |
|  | Valine | Val | 130.1 ± 3.3 | 89.5 ± 0.7 | 30.8 ± 0.7 |
